# Organ-specific alterations in tobacco transcriptome caused by the PVX-derived P25 silencing suppressor transgene

**DOI:** 10.1186/1471-2229-13-8

**Published:** 2013-01-08

**Authors:** Balaji Jada, Arto J Soitamo, Kirsi Lehto

**Affiliations:** 1 Department of Biochemistry and Food Chemistry, Laboratory of Molecular Plant Biology, University of Turku, Itäinen pitkäkatu 4B, 6. floor, PharmaCity, FI-20520, Finland

## Abstract

**Background:**

RNA silencing affects a broad range of regulatory processes in all eukaryotes ranging from chromatin structure maintenance to transcriptional and translational regulation and longevity of the mRNAs. Particularly in plants, it functions as the major defense mechanism against viruses. To counter-act this defense, plant viruses produce suppressors of RNA silencing (Viral suppressors of RNA silencing, VSRSs), which are essential for viruses to invade their specific host plants. Interactions of these VSRSs with the hosts’ silencing pathways, and their direct and indirect interference with different cellular regulatory networks constitute one of the main lines of the molecular virus-host interactions. Here we have used a microarray approach to study the effects of the *Potato virus X Potexvirus* (PVX)-specific P25 VSRS protein on the transcript profile of tobacco plants, when expressed as a transgene in these plants.

**Results:**

The expression of the PVX-specific P25 silencing suppressor in transgenic tobacco plants caused significant up-regulation of 1350 transcripts, but down-regulation of only five transcripts in the leaves, and up- and down-regulation of 51 and 13 transcripts, respectively, in the flowers of these plants, as compared to the wild type control plants. Most of the changes occurred in the transcripts related to biotic and abiotic stresses, transcription regulation, signaling, metabolic pathways and cell wall modifications, and many of them appeared to be induced through up-regulation of the signaling pathways regulated by ethylene, jasmonic acid and salicylic acid. Correlations of these alterations with the protein profile and related biological functions were analyzed. Surprisingly, they did not cause significant alterations in the protein profile, and caused only very mild alteration in the phenotype of the P25-expressing transgenic plants.

**Conclusion:**

Expression of the PVX-specific P25 VSRS protein causes major alterations in the transcriptome of the leaves of transgenic tobacco plants, but very little of any effects in the young flowers of the same plants. The fairly stable protein profile in the leaves and lack of any major changes in the plant phenotype indicate that the complicated interplay and interactions between different regulatory levels are able to maintain homeostasis in the plants.

## Background

RNA silencing is a highly conserved and versatile genetic surveillance and regulatory mechanism occurring in all higher eukaryotes. It is mediated by a large network of interacting effector molecules, and connected to several parallel regulatory and signaling pathways. It affects, both directly and indirectly, cellular processes varying from the cell cycle regulation and chromatin structure maintenance to transcriptional and posttranscriptional regulation, to stress and hormonal signaling, and to developmental differentiation [1-5]. Thus, RNA silencing plays a central role in manifestation of the genetic information of different eukaryotes. Many of the effector molecules of the silencing processes themselves are also regulated by specific silencing mechanisms, and many of the silencing-related regulatory pathways are interconnected in multidimensional networks [6,7].

The silencing process is induced by double stranded RNA (dsRNA) structures. These are recognized and cleaved into small fragments of 20–24 nucleotides by the RNase III type endonuclease Dicer in animals, or by various Dicer-like (DCL) homologues in plants. Depending on the source and form of the inducing dsRNA molecules, the products are called either microRNAs (miRNAs) or small interfering RNAs (siRNAs): miRNAs are cleaved from the hairpin structures of endogenous pre-miRNA transcripts, and they target specific complementary sites in their specific target transcripts. The siRNAs are cleaved from any nonspecific dsRNA molecules, and consequently target DNA or RNA sequences that bear homology to the inducing dsRNAs. In all cases, the small RNA fragments function as guide sequences for the silencing machinery: they are loaded into Argonaute (AGO)-containing effector complexes, i.e. into RNA-Induced Silencing Complexes (RISC), or into RNA-Induced Transcriptional Silencing Complexes (RITS), and guide these complexes to homologous RNA or DNA sequences to mediate their degradation or translational suppression, or methylation, respectively (reviewed in [2,4,8-10]).

The silencing networks are also essential in maintenance of cellular health and integrity. In animals, they play a major role in suppression of oncogeny [11,12], and in plants, they are involved with hormonal signaling and defense reactions against some bacterial pathogens [5,13-16]. In plants, RNA silencing is particularly used as the major defense mechanism against virus diseases (reviewed in [17-21]). Various virus-specific dsRNA structures (such as replicative intermediates, two-directional transcripts or local hairpin loops) function as efficient silencing inducers. These are processed to 21nt siRNAs by the DCL4 enzyme, or optionally by DCL2, and then mediate degradation of the homologous viral RNAs by the silencing machinery.

The virus-host interaction is further complicated by the production of virus-encoded suppressors of RNA silencing (VSRSs). Most, if not all plant viruses encode for at least one gene product that functions as a VSRSs. Many of the VSRSs mediate also some other essential viral function, e.g. several of them function as viral cell-to-cell or long distance movement proteins, coat proteins, replicases, helper components for viral transmission, proteases, or transcriptional regulators. Thus the VSRS produced by different virus families are different, with very different functional mechanisms [22-26]. For most VSRSs the exact modes of action are not well understood as yet, but obviously, through their interactions with the host silencing machinery (e.g. the small RNAs, DCLs or AGO proteins) they can strongly intervene with the regulatory pathways and networks of the host plants.

Several VSRSs have been expressed as transgenes in different host plants to study their interactions with the silencing machinery, and with plant endogene regulation. Some of these transgenes severely disturb the plant phenotypes and miRNA or mRNA expression profiles and affect their susceptibility to further viral infections, while some other transgenes have minimal or no effects on the plant phenotypes [27-31]. These differences obviously depend on the specific VSRSs, and possibly also on the specific host/VSRS combinations that have been used in these studies.

The P25 protein encoded by the *Potato virus X* (PVX) is a multifunctional protein that acts as both VSRS and cell-to-cell movement protein [32,33]. We have previously reported production and characterization of transgenic tobacco lines that express this VSRS gene [34], and here we report a microarray analysis (Tobacco 4×44K, Agilent) of the transcriptome of both leaves and flowers of these plants. The results indicate major changes in the transcript profile of the leaves, but only minor alterations in the transcripts of flowers of these transgenic plants.

## Results

**Figure 1:**
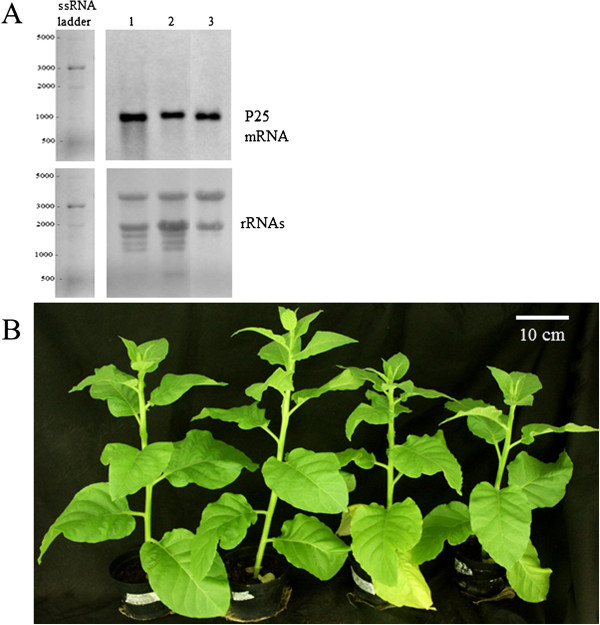
**Expression of the P25 transcripts in the leaves and in flower of the transgenic plants (A), and the phenotypes of the P25 expressing transgenic plants (B).** In A, the upper panel shows the Northern blot detection of the P25 mRNA from leaves (lanes 1 and 2) and from flower (lane 3) while the lower panel shows the ribosomal RNAs from the same sample lanes after methylene blue staining. Panel B shows two P25 expressing transgenic plants on the right, as compared to two wild type tobacco plants on the left. Through the early stages the transgenic plants show no difference in the phenotype, but the final size remains about 10% smaller than that of the wild type plants, and the lower leaves show slight premature senescence.

P25 VSRS –expressing transgenic tobacco plants (*Nicotiana tabacum* nn), along with tobacco lines expressing various other VSRS (e.g. *Potato virus Y potyvirus* specific HC-Pro and *African cassava mosaic* geminivirus specific AC2) have been previously produced and characterized in our laboratory [34]. We have earlier described a P25-expressing line with very low transgene expression level, that caused no detectable phenotype alterations in the plant, but for this study we have chosen a T3-line that accumulates P25 mRNA on higher levels. Expression level of the transgene transcript was equal in leaves and flowers of the same plants, as detected by Northern blotting and by quantitative RT-qPCR (Figure 1A and Additional file 1), but varied slightly between the sibling plants of the same T3-line (Figure 1A). As an altered phenotype, these plants showed a reduced growth, with height of the fully grown plants being about 10% lower than that of the wild type plants, and slightly enhanced senescence of lower leaves, as compared to wild type plants (Figure 1B).

Leaf samples (three biological replicates) were collected both from the transgenic and wild type control plants at about one and half months after germination, at plant height of approximately 20 centimeters. The P25-transcript levels were equal between these transgenic plants (Additional file 1). Our previous microarray analyses have revealed that the transcript profiles of control plants transformed with the empty pBin61 transformation vector are approximately equal to those of the wild type healthy plants [35], and therefore only the wild type plants were used here as controls. Flower samples were collected from the same plants one day before flower opening. Upon RNA isolation and quality controls the microarray analysis was done, and the obtained data analysed as described in the Methods, and by Soitamo et al. [35,36].

Many of the tobacco microarray probes are identified only by EST codes in the manufacturer’s specifications. Additional gene identifications for many of the positive microarray detections were obtained from the http://mapman.gabipd.org/web/guest/mapman-annotationexperts webpage. For those probes that were not found in this database additional annotation information was searched from the TAIR data base (http://www.plantta.jcvi.org/) and by the BLASTN- and BLASTX programs (NCBI). Initial functional grouping for all the identified, significantly altered transcripts was obtained from the MapMan database. However, many of the tentative functions could be associated with several biological roles, e.g., the genes related to disease responses may function also as transcription factors, in signaling, or can be related to cell wall modifications [37]. Therefore the functional grouping was manually adjusted to correspond to assumed gene induction during plant viral infection. Some related groups were also combined to reduce the total number of groups for clearer visual presentation.

**Figure 2:**
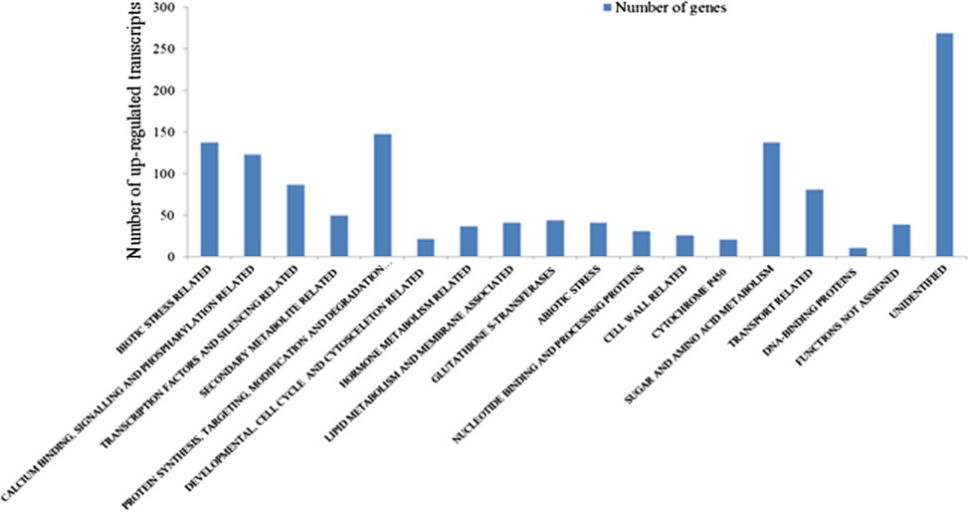
**Overview of the transcripts that are enhanced by 2-fold or more in the leaf samples of P25 expressing transgenic plants as compared to the wild type tobacco plants.** The functional groups of the up-regulated genes are shown in the x-axis and the number of transcripts in each group on the y-axis.

**Figure 3:**
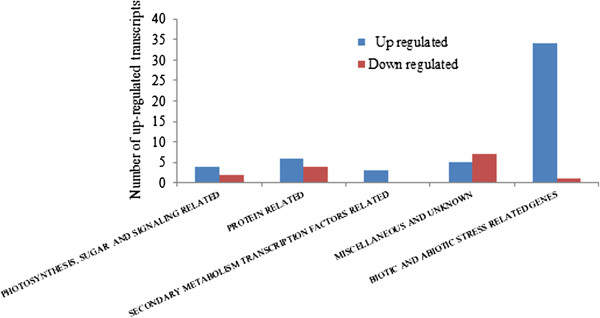
**Overview of the transcripts that are enhanced or reduced by 2-fold or more in the flower samples of P25 expressing transgenic plants as compared to the wild type tobacco plants.** The functional groups of the genes are shown in the x-axis and the number of transcripts in each group on the y-axis.

Using the two-fold cut-off-value for the up- and down-regulated genes and the stringent selection criteria of BH false discovery rate of 5%, the array data revealed that a total of 1350 (non-redundant) spots produced a significantly (with adjusted *p* value < 0,05) up-regulated signal from the leaf total RNA (Figure 2). A full list of all detected up-regulated genes is given in Additional file 2. The main types of the up-regulated genes detected in each functional group, and the range of their amplification levels are also listed in Additional file 3. Using the same stringent criteria only 5 transcripts of the leaves were found to be reduced to the 0.5-fold or lower level. 51 and 13 transcripts were found significantly up-regulated and down-regulated, respectively, in the flowers of the P25 expressing plants as compared to the wild type plants (Figure 3, Additional files 4 and 5).

Selected up- and down-regulated genes were analyzed by the quantitative real time PCR (RT-qPCR) to verify the array data for both leaf and flower samples of the transgenic vs. wild type plants. The RT-qPCR results correspond approximately to the microarray results obtained for these genes (Table 1).

**Table 1:** Microarray results verification by using quantitative real-time PCR (RT-qPCR)

**Leaf (up regulated transcripts)**		**Microarray**	**RT-qPCR**	
**EST/mRNA**	**Description**	**Log2**	**Log2**	**s.e.**
EH617029	Capsicum annuum; WRKY transcription factor-30	5.87	5.71	0.15
EB438730	Dicer-like 2 splice form	2.09	2.17	0.16
EH620111	Pathogenesis-related protein 1B precursor	10.7	14.5	0.53
**Flower (up regulated transcripts)**				
EH620111	Pathogenesis-related protein 1B precursor	4.0	5.71	0.51
Z11563	Acidic endochitinase precursor	2.53	2.75	0.41
**Unchanged transcripts**				
EB450395	ARPC3 (actin-related protein C3)	−0.04		
AM833694	DCL-1 (Dicer like 1)	0.003		

Expression levels of some clearly up-regulated genes were tested by RT-qPCR. Statistical significance was tested using student t-test (p<0.05), with BH value of < 0.05. The depicted log2 values are normalized median intensive value (n=3) differences of wild type and P25-expressing transgenic plants. The Standard error of the mean (s.e.) is calculated for the C_t_ values of the RT-qPCR results.

As tobacco genome has not been sequenced, the annotation of the significantly up- or down-regulated genes were mostly based on similarities to known genes or to known functional domains in other species, and thus the names, alleles or biological functions could not be accurately determined for all the detected transcripts. In many cases there were also more than one probes, with different EST-codes for the same RNAs. Thus, the array data revealed only the number of altered hybridization events within the given set of 44000 probes, but did not provide accurate numbers for the actual up- or down-shifted genes. Still, the repetitive detections of the same genes, their multiple alleles, multiple members of the gene families, or of genes belonging in the same pathways confirmed the alterations in the expression levels of these genes in the transgenic plants.

### Up-regulated transcripts related to biotic and abiotic stress in leaves

**Figure 4:**
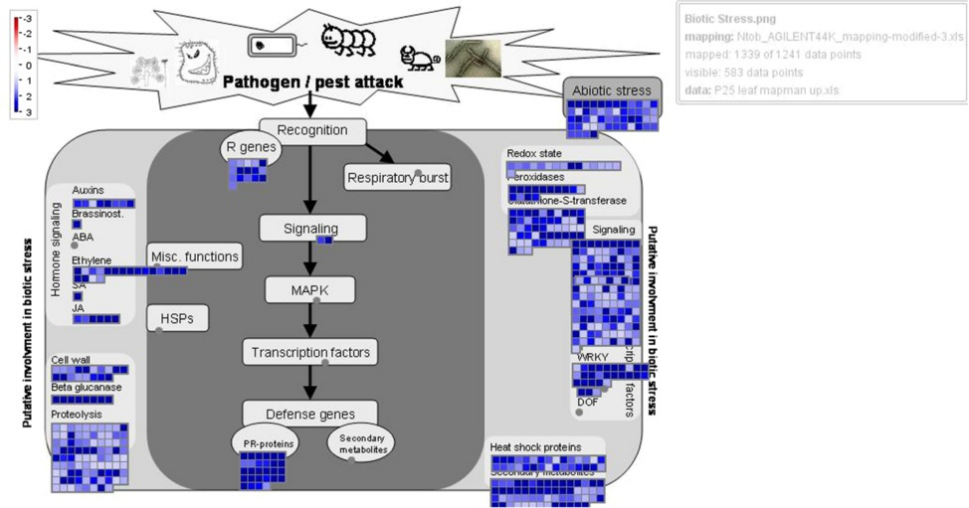
**Up-regulation of biotic stress related genes (log2 value >1) shown by using the MapMan software.** The blue squares represent the number of up-regulated transcripts that are involved in biotic stress reactions. The pathway description and visual presentation of involved genes are described in [38].

The annotated and functionally categorized microarray data revealed that the P25-protein expressing plants were primed for a strong pathogenesis-like condition [37]: a total of 138 up-regulated transcripts belonged to the genes that are induced by various biotic and unspecific stresses (Figure 2 and 4, Additional files 2 and 3). This group included, for instance, a total of 30 genes coding for various Pathogenesis-related proteins (of types 1a, b and c, 4b, R, and PX). Many of these were very strongly induced, even up to three thousand folds, which rate is also explained by their very low or undetectable expression level in the healthy control plants. Also various disease resistance genes (a total of 21), and genes coding for Avr9/Cf-9 and Hairpin elicited proteins (21 detections), various chitinases and endochitinases (17), systemic acquired resistance (SAR) -associated proteins (19) and hypersensitive cell death (HR) –associated proteins (10) were strongly induced. Interestingly, this analysis detected also enhanced expression of a TMV-response (*N*-gene) related transcript, although this tobacco variety is of the *nn* type and does not contain the functional *N*-gene. Thus, the non-functional allele (*n*) is induced in a similar fashion by disease stresses as is the functional resistance gene *N*.

Enhanced stress and disease resistance status was also indicated by the strong enhancement of various transcripts (total of 50) related to biosynthesis of secondary metabolites, with maximal, about 200-fold enhancement occurring in the epi-aristolochene synthase. Transcripts coding for the phytoalexins and various flavonoids, cinnamoys and terpenoids that may serve as constitutive or inducible chemical inhibitors of various pathogens [39] were also enhanced. Of special interest is the enhanced expression of REF/SRPP-like isoprenoids, as these are needed for miRNA function and affect membrane association of AGO1 [40].

In addition to the enhanced response to biotic stresses, also various indicators of abiotic stresses were induced (total of 41 detections), although not as strongly as those for the biotic stresses. The highest induction levels in this category were those for the metal binding protein genes (77-fold) and the oxidative stress genes (11-fold enhancement). In addition, a total of 26 transcripts of various heat shock proteins and chaperones were enhanced by 2 – 7 -folds. Other up-regulated stress genes were associated to dehydration/desiccation, osmotic stress and senescence.

### Up-regulation of transcripts related to protein synthesis and modification, and to metabolic networks in leaves

Many transcripts coding for the protein synthesis machinery, protein transport and processing, energy metabolism, lipid metabolism, membrane-associated proteins and molecular transporters were also strongly altered in the P25-expressing plants (Figure 2, Additional file 3). A total of 9 transcripts related to transcription or translation initiation or to the translation machinery were increased by 2–33 -fold suggesting that, in addition to the increase of various transcript levels, also the protein synthesis was enhanced. Maybe to balance this disturbance, also a large number (total of 110) of transcripts associated with the ubiquitin-mediated protein degradation, or encoding various proteases, endopeptidases, carboxypeptidases, hydrolases and numerous AAA-type ATPase transcripts were enhanced to variable extent (by 2 – 180 -fold) (listed under groups “Protein synthesis, targeting, modification and degradation related” and “Nucleotide binding and processing proteins” in Additional files 2 and 3; see also Additional file 6).

Multiple changes were detected in the sugar and amino acid metabolism related transcripts: Photochemistry-related signaling was altered by strong induction of two PAR–transcripts and of three ethylene induced genes. In addition, a total of 133 transcripts coding for various sugar metabolism-related genes and 5 transcripts coding for components of the mitochondrial electron transfer chain were enhanced in the range of 4 – 39 -fold.

### Up-regulation of membrane and lipid metabolism and of molecular translocation in leaves

A total of 15 transcripts coding for various membrane proteins were enhanced by 2 – 12-fold. In addition, 26 transcripts related to various membrane or lipid modification activities were enhanced, some up to 94-fold (Additional file 3). Also 27 transcripts associated with various cell wall modification activities were enhanced, including transcripts for cell wall degrading enzymes xyloglucan endotransglucosylase–hydrolases (9 transcripts), and glucan endo-1,3-beta-glucosidase (6 transcripts). Of special interest is the enhancement of transcripts for Pectin methyl esterase (PME) inhibitor-type proteins which, by their action on PME may contribute to silencing suppression [41].

Transport and translocation of various metabolites appeared to be disturbed in these transgenic plants as nearly a hundred transport- and secretion-related transcripts were enhanced: 19 transcripts related to protein secretion to various membranous compartments (ER, vesicles, peroxisomes, nucleus) were enhanced up to 33-fold. 17 transcripts coding for various amino acid and oligopeptide transporters, and 21 transcripts coding for transporters for various phosphates, sulphates, ammonium ions, metals and nitrates were also enhanced, as were eight transcripts for ion channels and seven transcripts for sugar transporter, and a total of 15 miscellaneous transport related transcripts. Enhanced abiotic stress appeared to be associated with the induction of 13 transcripts encoding for different multidrug resistance proteins, or ABC transporters.

In addition to the mentioned functional groups, also 11 transcripts related to DNA binding, and 6 transcripts related to cytoskeleton were induced up to 10–fold in these plants. Also 13 transcripts coding for proteins with a DUF-domain, i.e. domains with unknown functions [42], and a large number of transcripts with no clear functional identification (a total of 39), as well transcripts with no identification of the coding sequence (256) were significantly enhanced in the transgenic leaves (Figure 2, Additional files 2 and 3).

### Regulatory factors for enhanced gene expression in leaves

Induction of the large battery of the defense-related genes is clearly associated with strong induction of the signaling factors that serve as primary regulators of the defense pathways. Typically, plant defense responses are induced by salicylic acid (SA), ethylene (ET) and jasmonic acid (JA) [43]. Induction of these regulatory pathways (Figure 4) in the P25-expressing plants was indicated by strong (up to 8-fold) enhancement of two transcripts coding for one key signaling factor in the SA-induced pathway, i.e. the Enhanced disease susceptibility (EDS1) factor [44]. Likewise five transcripts coding for 1-aminocyclopropane-1-carboxylate (ACC) synthase, oxidase and deaminase, and two transcripts coding for 2-oxoglutarate-dependent dioxygenase, all involved in the ethylene biosynthesis [45] were strongly enhanced, even up to 95-fold. Several transcripts for ethylene induced signaling or transcription factors (e.g. ER4, ER2) were enhanced by up to 40-fold. Eight transcripts coding for membrane–degrading lipoxygenases, leading to JA synthesis [46] were enhanced by up to 5-fold. Also several ethylene responsive (9), auxin responsive (6), and gibberellin related (a total of 5) transcripts were induced. Ethylene response transcription factor 1 (ERF1), i.e. the key signaling factor which integrates signals from ET and JA signaling pathways [47], and ERFs 3 and 5 (of unknown function) were enhanced by 5–25 -folds.

Further on, numerous transcripts (total of 79) for different transcription factors, some of them known to be regulated by ERF1 or by JA [47], were enhanced in the P25-expressing plants. These included 29 transcripts for WRKY domain proteins, and multiple transcripts for PTI5, RAV, NAC and MYB-like transcription factors, some of which are known to be transcriptional regulators of various disease responses [48-50].

The enhanced transcription factors included also several that function as developmental regulators and are typical targets for silencing-mediated regulation, i.e. the SCARECROW, AP2 and GRAS –type transcription factor with the DELLA domain, WAF related cluster (a hypothetical protein belonging in the Auxin response factor family), the floral identity gene LEAFY [51], and multiple transcription factors containing the Nam (No Apical Meristem) or NAC domains, known to be involved in many developmental processes as well as in defense reactions [52]. Also chromatin methylation associated Histone Deacetylation (HD2) gene and High Mobility Group B 3 transcription factor gene were up regulated (Figure 2 and Additional file 2). Enhancement of four microsatellite DNA transcripts (listed under “DNA binding protein”) as well as of five retro element-related sequences (listed under “Biotic stresses”) suggested reduced methylation level of the genomic DNA.

Of special interest among the induced RNA-related regulatory mechanisms was the induction of the DCL2 enzyme, known to serve as the alternative effector molecule, in addition to DCL4, in silencing of viral RNAs [53], of AGO2, known to function in virus-defense-related silencing [54], and of RNA dependent RNA polymerase 1, known to function as an endogenous silencing suppressor [55].

Calcium and phosphorylation mediated signaling and regulatory networks were also altered (Figure 2, Additional file 7). A total of 115 transcripts related to protein phosphorylation and Ca-mediated signaling were enhanced, including various Ca-binding proteins and Calmodulins (35 transcripts), e.g. the rgs-Calmodulin, the endogenous silencing inhibitor protein [56]. Enhanced signaling-related genes included also various kinases (30) and receptor kinases (32), as well as some phosphatases. Of special interest is the induction of several (7) MK1 and MAP3 kinases, and MAP kinase kinases (2), known to mediate the signaling cascade that leads to transcriptional activation of various defense genes, e.g. some WRKY transcription factors [43].

In these transgenic plants, a total of 44 transcripts coding for various Glutathione-S-transferase enzymes were enhanced by up to 80-fold, and a total of 21 transcripts coding for various Cytochrome P450 or closely related enzymes were enhanced by up to 63-fold. These enzymes are known to function in cells as antioxidants, and to detoxify and degrade endogenous compounds such as peroxidased lipids and various harmful metabolic intermediates and toxins [57].

### Down-regulated transcripts in leaves

As mentioned earlier, only five transcripts (Heparan-alpha-glucosaminide N-acetyltransferase, hydrolase, two-component response regulator-like APRR5, and two unknown transcripts) were found to be significantly reduced, by applying the stringent selection criteria of BH false discovery rate of 5%. By neglecting this stringency test some 325 transcripts were also found to be reduced to 0.5-fold or lower level, as compared to the wild type plants, but these reductions were much more moderate and inconsistent than the up-regulation of various transcripts (data not shown). The most interesting group among these mildly down-regulated transcripts was that of the photosynthesis-related functions, including the photosynthesis-related Calvin cycle transketolase, light reaction-related ATP-synthase and a post-illumination chlorophyll fluorescence increase protein (related to cyclic electron flow), photosystem I assembly and stability factor, and several transcripts for Phototropic-responsive NPH3 family proteins functioning in light signaling [58].

### Altered gene expression in the flowers of P25 expressing plants

From the samples extracted from unopened flowers, only 51 transcripts were detected as significantly up-regulated and 14 as significantly down-regulated in the microarray analysis. As in leaves, the most significant up-regulated functional group was that of transcripts associated with various biotic stresses, including 15 transcripts coding for different pathogenesis-related proteins, 9 transcripts coding for various chitinases and endochitinases, and 4 transcripts coding SAR-related proteins. The up-regulated transcripts included also the Photosystem II 10 kDa protein and Rubisco small subunit proteins, up-regulated by 4- and 3- fold, respectively. Up-regulated genes included also e.g. one glutathione transferase, two vetispiradiene synthase and three epoxide hydrolase transcripts (Figure 3, Additional files 4 and 5).

The transcripts that were found to be down-regulated in flowers included three transcripts for methylthioadenosine nucleosidase, three transcripts for glycine-rich proteins, as well as a transcript for photosystem I reaction center subunit PsaN (Figure 3, Additional files 4 and 5).

### Alterations in the protein profile in leaves of P25 expressing plants

**Figure 5:**
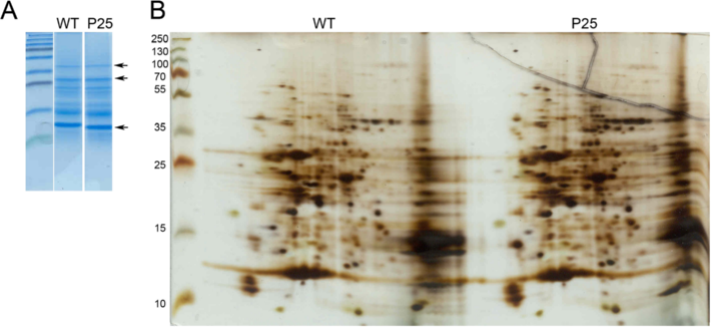
**1D-SDS-PAGE gels (A), and 2D-polyacrylamide gel electrophoresis (2D-PAGE) (B) to show, respectively, the equal loading of samples and the levels of various individual proteins.** 1D gels are stained with coomassie blue, and the 2D-gels using silver staining kit. Several other blue-stained 2D-gels are shown in Additional file 8.

Major changes observed in the accumulation of various mRNAs in P25-expressing plants, including the mRNAs coding for some key components of the translational machinery (particularly for different translation initiation factors) and also in the protein degradation machinery (see Additional file 6) suggested that the total protein content or the protein profile of the plants might be significantly altered. However, the total protein content of the P25 expressing plants was found to be equal to that of the wild type plants, measured as weight of soluble protein per fresh weight unit of the leaves. Also the protein profile, as revealed by 2D-PAGE from the phenol extracted samples was very similar to that of the wild type plants (Figure 5). In repeated analysis of protein samples (total of 5) extracted from the third leaf of different plants we sometimes observed increase of several protein spots (Figure 5 and Additional file 8), and sometimes a clear reduction of multiple spots, but these changes were not consistent in all the gels and thus we conclude that there was no significant alteration in the protein profile of the P25-expressign plants, as compared to the wild type plants.

### Photosynthetic activity

**Figure 6:**
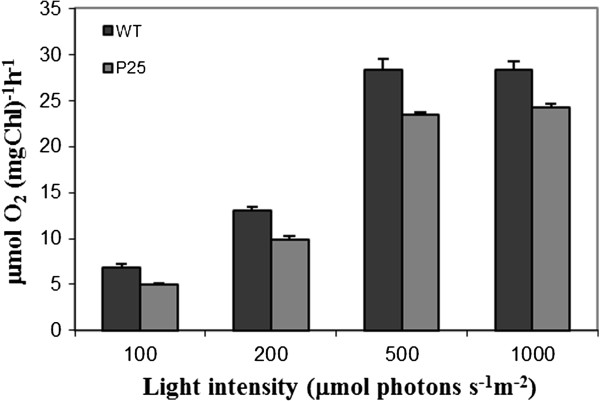
**Light-responsive O**_**2**_**-evolution of photosystem II was measured of wild type and P25 expressing plants.** O_2_-evolution was measured of freshly isolated thylakoid membranes using DCBQ as an electron acceptor. Four light intensities is shown in x-axis (μmol photons s^-1^ m^-2^) and the O_2_-evolution in y-axis (μmol O_2_ (mg Chl)^-1^ h^-1^). Standard error of mean is presented as bars above the columns (n = 4, consisting of four biological replicates).

To analyze the overall physiological condition of the transgenic plants, as indicated by their photosynthetic activity, their O_2_ evolution activity was determined from freshly isolated thylakoid membranes at different light intensities. The measurement indicated that the photosynthetic activity was reduced by approximately 20% in the transgenic leaves, not depending on the light intensities (Figure 6). Interestingly, this reduction is the same as has been earlier reported for tobacco plants infected with the PVX virus [59]. Here, it was apparently related to the mild down-regulation of several photosynthesis-related transcripts (ATP synthase protein I, transketolase, photosystem I assembly and stability factor) in these P25-expressing leaves.

### Responses to biotic and abiotic stresses

**Figure 7:**
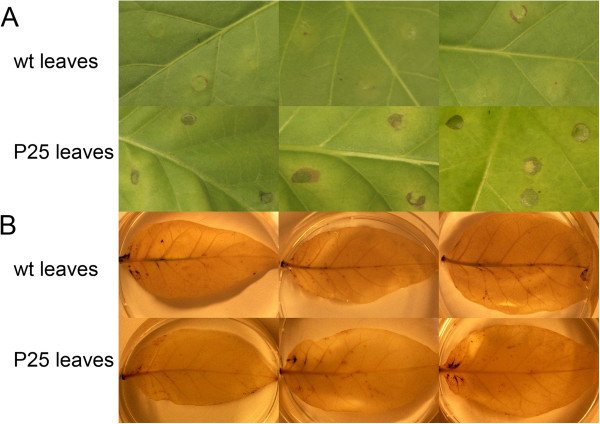
**Responses in leaves of wild type (upper row) or of P25 expressing transgenic tobacco plants (lower row) caused by the infiltration of Pseudomonas syringae pv. tomato DC 3000 cell suspension (A) and detection of reactive oxygen species in untreated leaves (B).** In wild type leaves (upper row) the infiltrations with P. syringae cells caused no visible reaction, or occasionally, a mechanical damaged ring at the site of the syringe contact, while in P25-epressing leaves (lower row) they caused clear HR reaction at the infiltration site (**A**). Detached leaves of the wt (upper row) and P25-expressing leaves (lower row) show very similar DAB and NTB-staining pattern, indicating that the transgenic leaves do not contain any additional oxidative compounds.

The massive induction of various transcripts related to active defense against various plant diseases (Avr9/Cf-9 elicited transcripts, PR-proteins, chitinases, endochitinases, disease resistance genes, HR-associated proteins, SAR-related proteins) suggested that the plants should be primed for enhanced resistance against various pathogens [43]. This was tested by infiltrating the leaves with a suspension of *Pseudomonas syringae* pv. tomato DC 3000 cells. These cells were avirulent in the wild type tobacco leaves, causing either no, or only very minor hypersensitive reaction in the infiltrated leaf zones. When infiltrated to P25-expressing plants, they caused clear HR-response within 6 days after infiltration (Figure 7A).

High expression levels of transcripts coding for various peroxidases, oxidases, reductases and oxidoreductases suggests that these factors might be associated with high oxidative stress in the leaves. The existence of such stress was tested by DAB and NTB-staining of the leaves [60]. The P25-expressing leaves produced essentially the same staining pattern as the wild type leaves, with only minor background darkening, indicating that the plants did not contain any additional oxidative compounds (Figure 7B).

### Genes commonly induced with the HC-Pro and AC2 silencing suppressor expressing plants

**Figure 8:**
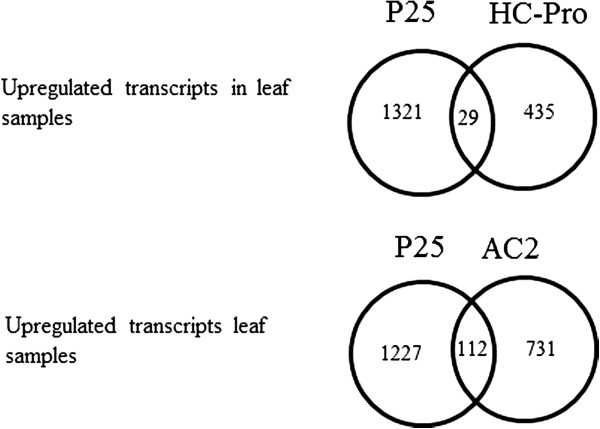
A Venn diagram presents the number of unique and commonly up-regulated genes in between leaf samples of P25, HC-Pro and AC2 expressing transgenic tobacco plants.

The transcripts enhanced in the P25-expressing plants were compared to the genes that have been found to be enhanced in other transgenic tobacco lines, expressing either the PVY-derived VSRS HC-Pro, or ACMV-derived VSRS AC2. Although large number of gene expression alterations have been observed also in the HC-Pro and AC2 expressing leaves (748 and 1118, respectively), the alterations patterns were distinctly different between these three types of plants, with only 137 common transcripts being enhanced in all of these transgenic plants (Figure 8, Additional files 9, 10, 11). The commonly induced transcripts included transcripts related to plant defense against biotic and abiotic stresses (e.g. EFE enzymes for ET production, Ethylene-responsive transcription factor 1, MAP3 kinase, Glutathione S-transferase, Cytochrome P450, some SAR-related genes, WRKY transcription factors, chitinases and endochitinases, Calmodulin-like protein, and several transcripts related to oxidative stress). Also some transcripts associated with cell wall modifications (xyloglucan endotransglucosylase-hydrolase) and transcripts related to transport, senescence and ripening, secondary metabolite biosynthesis and oxidative stress were induced in all these transgenic plants (Additional files 9, 10, 11).

## Discussion

Here we report microarray (Tobacco 4 × 44k, Agilent) mediated analysis of the transcriptomic profile of transgenic tobacco plants that express the P25 RNA silencing suppressor of the PVX virus. According to the microarray results, this transgene caused a significant up-regulation of numerous (a total of 1354) transcripts, with induction levels ranging up to 3000-fold level. This up-regulatory effect, as compared to the low number of significantly down- regulated genes (a total of five, as determined by the same stringent selection criteria) indicated that the transgene caused active silencing suppression in the tobacco plants. A special feature of these gene expression alterations was that they were detected mostly in the leaf samples. In flowers, the levels of only a few transcripts (a total of 64) were slightly altered.

The altered gene expression profile was associated only with mildly reduced growth, and occasionally, premature yellowing of the lower leaves, which apparently correlated to the slightly reduced photosynthetic activity and the enhanced ethylene expression in these plants. This phenotype is in good agreement with previously published phenotype of P25-expressing tobacco plants [61]. Also the observed reduction of the photosynthetic rate was the same as what has been reported for tobacco plants infected with PVX virus [59], indicating that the expression of the P25 VSRS-protein is the main cause of the yield reduction also in the virus-infected plants.

Surprisingly, the plants did not show any major phenotype alterations although the up-regulated transcripts included several developmentally important transcription factors, such as those coding for the SCARECROW, LEAFY1, AP2 and GRAS/DELLA –type, or NAC, Nam (No Apical Meristem), MYB and MADS domain containing transcription factors, or the WAF-protein of the Auxin response factor family. Also several development-related genes coding for embryo-abundant, senescence associated, ripening responsive or storage proteins were strongly enhanced.

The most significant groups of the up-regulated genes were those associated with various biotic and abiotic stresses, including numerous disease resistances, pathogen induced and systemic resistance (SAR) related transcripts, as well as transcripts for heavy metal, draught, and osmotic stress tolerance. These appeared to be induced in a coordinated fashion, via strong induction of the biosynthesis of their key signaling factors, i.e. SA, ET and JA [44,45]. Induction of these regulatory pathways via silencing suppressor function indicated that they are regulated via RNA-silencing, as previously shown for JA [62]. The strong activation of the multiple disease defense genes in these plants was associated with a clearly enhanced HR reaction to invading bacteria, as compared to wild type plants.

One important effect of the P25 protein was the strong enhancement of numerous signaling-related transcripts, which all play a significant role in regulation of gene expression and in various enzyme activities. Important examples of these are the MAP3, MK1 and MAP kinase kinases which mediate the signaling cascade downstream of the primary defense response factors (Figure 4) [43]. Of special interest in this group is the enhancement of the transcript of the rgs-CaM gene which itself acts as a suppressor of RNA silencing in the tobacco [56]. Its up-regulation in these transgenic plants raises the possibility of its involvement in the silencing suppression mechanism of P25 VSRS.

Surprisingly, the strong induction of multiple transcripts did not affect the level of total soluble protein content, and no significant or consistent alterations were either observed in the protein profile of the transgenic plants, as compared to wild type plants (Figure 5 and Additional file 8). Apparently the general enhancement on transcript accumulation was effectively counter-balanced by increased protein degradation, as indicated by the strong increase of multiple transcripts related to different proteolytic pathways (Additional file 6). Lack of any distinct phenotype in the P25-expressing plants is consistent with the (fairly) stable protein profile of the plants, and these both indicate that the homeostasis in the plants is maintained via complicated interplay and interactions between different regulatory levels. The enhanced level of multiple ubiquitin-related transcripts indicates that the regulatory network involves enhanced proteosomal degradation of proteins (see Additional file 6). Proper proteosomal function has been shown to be essential for the maintenance of plant health and homeostasis [63].

In contrast to the stable phenotype of the P25-expressing plants, two other VSRS-transgenes (HC-Pro and AC2) cause severely altered phenotypes in transgenic tobacco plants [34-36]. Interestingly, alterations observed in the transcriptomes of the P25-expressing plants were quite different from those observed either in the HC-Pro or AC2 VSRS expressing transgenic plants, as P25 caused predominantly only up-regulation of the transcripts, and affected much larger number of transcripts, with an overlap of only 138 altered transcripts between these three VSRSs. This comparison reveals that some of the genes that were differently affected in these lines were the causal factors for the severe developmental disturbances and phenotypes observed in the HC-Pro and AC2 expressing lines.

### Functional mechanisms of the P25 induced effects

P25 VSRS is known to interfere with the AGO1, the central component of the RISC complex, and mediate its degradation through the proteosomal pathway [64]. Defects of the proper AGO1 function or expression are known to compromise all DCL1, DCL2 and DCL4 mediated silencing activities, and depending on the type of *ago 1* mutant allele, may severely disturb the development of the plant [65]. In our transgenic plants the expression of P25 – with assumed disturbance of the AGO1 protein – had a strong effect of the transcriptome, but hardly any effect on the plant phenotype. This may be related to previously reported phenomenon that the *ago1* mutants affect the silencing of some, but not of all of miRNA-target genes [66]. Also in our P25-expressing transgenic plants, strong up-regulation was observed in some silencing-targeted genes e.g. in the defense-, signaling- and metabolism-related target transcripts, and in transcripts of many transcription factors with NAC, Nam, LEAFY, MYB or SCARECROW domains, while no alterations occurred in other typical silencing targets, such as various Auxin response factors, DCL1 or AGO1, or in other transcription factors that regulate the developmental differentiation [51].

AGO1 itself is known to be regulated by silencing–mediated pathways, and particularly via the function of the miR168. This effector molecule, as well as AGO1-derived siRNAs and their amplification loop mediated by DCL2, DCL4, RDR6, SDE5 and SGS3 are known to maintain the homeostasis of the AGO1 protein [67]. Another VSRS, i.e. the P38 derived from *Turnip crinkle virus*, also disturbs the AGO1 [29]. Likewise with the P25, this VSRS does not induce any specific phenotype alterations when expressed in transgenic *Arabidopsis* plants, although it - unlike with P25 - causes major disturbances to the silencing machinery via strong enhancement of the DCL1 and consequent (DCL1 mediated) suppression of DCL 3 and DCL4 [29]. In that case, the regulatory interactions reveal an intensive regulatory network that serves to maintain the homeostasis of the silencing machinery [68,69]. In the P25 expressing transgenic plants such network was not induced, as the expression levels of the DCL1, DCL3 or DCL 4 were not changed, revealing no explanation for the stabilizing mechanism in these plants.

One interesting feature is the tissue-specific regulation of the AGO1-mediated silencing pathways, as widely discussed by Voinnet [2]. It has been reported that the *ago1* mutants do not cause defects in apical meristems [70], and thus the P25-specific silencing suppression effect, mediated via AGO1 suppression, may not be effective in this tissue. Also the work of Faivre-Rampant and co-workers [71] suggests that PVX-P25 protein functions in an organ-specific fashion, with different silencing suppression effects in leaves and tubers. In our study, the P25-transgene had massive effects on the transcriptomes of the leaves, but minimal effects in the flowers, although the gene was expressed on equal level in both organs. Particularly strong silencing-mediated regulatory and surveillance mechanisms are known to operate in the plant meristems [72], and it is possible that the tissue-specific mechanisms prevent the disturbance of the AGO1 function in this tissue, to maintain the developmental integrity of the plants. The balancing mechanisms that modulate the AGO1 function and maintain cellular homeostasis in different organs need further investigation. The question of the regulatory mechanism(s) that are responsible for the plant homeostasis and the maintenance of the stable protein profile in the P25-expressing plants remains open.

## Conclusions

Results of this study indicate that the expression of the PVX-derived P25 VSRS in transgenic tobacco plants leads to enhanced accumulation of numerous transcripts (total of 1354) in the leaves, but changes the levels of only a few transcripts in the flowers of these plants. In spite of the massive changes in the transcriptome, the protein profile of the leaves remains fairly stable, and no major changes are observed in the phenotype of the plants, except for slightly reduced growth and the strong induction of biotic and abiotic stress indicators. Based on the alterations observed in different transcripts, we conclude that the maintenance of homeostasis in these plants involves enhanced proteosomal degradation of proteins. Lack of phenotype alterations may also relate to the organ-specific effects of the P25 VSRS, which occur in leaves but not in flowers. It is possible that the developmentally sensitive meristematic tissues are not venerable for these effects.

## Methods

### Plant material

Transgenic *Nicotiana tabacum* lines expressing the VSRS P25, HC-Pro and AC2 have been previously produced and characterized in our laboratory [34-36]. The plants were grown in normal growth conditions, with 150 μmol photons m^-2^ s^-1^, at 60% RH with a 16 h / 8 h light / dark cycle, at 22°C. Leaf samples (whole third leaf from the top) were collected from both wild type and P25 expressing transgenic tobacco plants at 6–7 weeks after germination. Flower bud samples were also collected from the same plants, just prior to the bud opening. Both leaf and flower samples were frozen in liquid nitrogen and stored at −80°C for further analysis.

### RNA extraction, cDNA labeling, microarray hybridization and analysis methods

Total RNA was extracted from both wild type and transgenic P25-expressing tobacco plants by using TRIsure reagent (Bio line, UK) according to manufacturer’s instructions. Then extracted total RNA was purified with the Nucleospin RNA purification kit followed by DNaseI treatment (Promega RQ1 RNase free-DNAseI) according to the instructions. The total RNA was concentrated by using Amicon Ultra-0.5 centrifugal filter devices. Upon RNA isolation, the RNA quality controls, the cDNA labeling and microarray hybridization were performed according to Agilent’s standard procedures, as stated in Soitamo et al. 2011 [35]. The obtained microarray data was normalized across the three biological replicates. The gene expression data was analyzed using the Chipster (CSC, Espoo, Finland) software, by comparing the means of three biological replicates of the wild type and the P25-expressing plants, by using Student’s t-test with the Benjamini and Hochberg (BH) false discovery rate of 5%, to determine whether their expression levels differ significantly (adjusted p-value < 0.05).

### Re-annotation of Differentially Regulated Gene Elements

The probe information for the Agilent microarray chip is based only on EST and cDNA sequences and thus is very limited. Additional, fully descriptive annotation information for many of the probes was obtained from the MapMan website [73,74]. For those probes that were not found in this data base, additional annotation information was searched on the JCVI website http://plantta.jcvi.org/. The positive detected transcripts were first categorized to functional groups based on the functional grouping available on the MapMan website, but the grouping was manually adjusted to some extent, to combine various metabolism-related groups and to assign stress-related genes to their own specific groups.

### Verification of differentially expressed genes

The quantitative real-time PCR (RT-qPCR) method was used for verifying the microarray results according to the MIQE guide lines [75] (Additional file 12). The cDNA synthesis was done by using 1ug of purified total RNA from both leaf and flower samples with the Revert Aid reverse transcriptase (product # EPO441, Fermentas). The produced cDNA samples were diluted 1:15 with sterile MQ-water. The RT-qPCR samples were made up with 10 ng (3 μl) of diluted cDNA samples, gene specific primers and Maxima SYBR Green/Fluorescein RT-qPCR Master Mix (2X) (Product #K0242, Fermentas) according to the manufacturer’s recommendations. To minimize the pipetting errors, 3–4 technical replicates were performed for each biological replicate. The RT-qPCR was performed by using Bio-Rad iQ5 machine in 96–well plate. The results were calculated based on the quantification cycle (Cq) method (delta delta Cq). Primers specificity was controlled by examining the single peak in their DNA melting curves. The standard error of mean was also calculated from three biological replicates.

### Isolation of proteins, 2D-PAGE

Protein samples were isolated from the same samples that were used as for RNA extraction, or, for additional samples, the samples were collected in the same way, from the third leaf from the top, and using the whole leaf. Samples collected from the wild type and P25 transgenic plants were first extracted by using TRIsure reagent (Bio line, U.K) with a protocol adapted from TRIzol (Invitrogen Inc. USA), as for the RNA isolation. In brief, the phenol phase was first washed with 100% ethanol (in ratio 0.3 ml EtOH / 1 ml phenol) and then the total proteins were precipitated from the phenol phase with isopropanol (isopropanol added in ratio 1,5 ml isopropanol/1 ml phenol). After centrifugation (12000 g, 10 minutes) the pellets were washed three times with 4 ml of 0.3 M Guanidine hydrochloride, made in 95% EtOH, by incubating at RT for 10 min, and with centrifugation at 7500 g for 5 min. The pellets were washed two times with 100% EtOH, dried, and solubilized in 8M Urea in 10 mM Tris/HCl, pH 7,5. The protein concentration was measured by using Bradford method (Bio-Rad protein assay kit) and a total amount of 250 μg of leaf protein sample both from the wild type and P25 transgenic plants was used to analyze the protein profiles. First the proteins were separated by isoelectric focusing using Bio-Rad 7 cm IPG, pH 3–10 strips, followed by second dimension separation in PAGE gels with the protein II apparatus (Bio-Rad). The resulting protein gels were fixed and incubated with coomassie blue stain (Page Blue staining kit, Fermentas) overnight, destained and photographed. Later the gels were stained again with silver staining kit (PAGE silver staining kit, Fermentas) according to manufacturer’s instructions and photographed.

### Photosynthetic Measurements

The equal amount of leaf sample (1.0 g, taken from the third leaf from the top) from wild type and P25-expressing transgenic plants were taken and ground in the 4 ml of thylakoid isolation buffer (0.3 M sorbitol, 50mM HEPES/KOH pH 7.4, 5mM MgCl_2_, 1mM EDTA, and 1%BSA) by using an ice cold mortar. The suspension was filtered through Miracloth and 2 ml of the resulting filtrate was pelleted at 12000 × g centrifugation for 2 minutes. The supernatant was removed and the remaining pellet was resuspended in oxygen electrode buffer (0.3 M sorbitol, 50mM HEPES/KOH pH 7.4, 5mM MgCl_2_ and 1mM KH_2_PO_4_). The oxygen evolution measurements were carried out by a Clark type electrode using 0.5mM DCBQ as electron donor. The chlorophyll concentration measurements were done according to the procedure stated in [35].

### HR response and oxygen radicals detection in leaves

6–7 week old plants were used for the study of the hypersensitive reaction (HR) in the P25 transgenic plants and in wild type plants against *Pseudomonas syringae* pv. tomato DC3000 infection. Freshly grown bacterial cells, suspended in the 10mM MgCl_2_ solution, were gently infiltrated (about 50 μl/spot) from lower side of the leaves into the intracellular space by using a syringe. Plants were incubated in normal growth conditions, and after a week the infiltrated leaves were photographed.

Hydrogen peroxide and superoxide radicals were detected by staining in 3,3'-diaminobenzidine (DAB) solution (0.1mg/ml DAB in MQH_2_O, pH 3.8 adjusted by NaOH) and in nitroblue tetrazolium (NBT) staining solution (0.1 mg/ml NBT in 25mM HEPES/KOH pH 7.4) respectively [60]. Leaf samples were collected and submerged into the staining solutions, and kept for the overnight in dark incubation. Next morning, leaf samples were treated with 96% ethanol approximately for one day to remove the chlorophyll and photographed.

## Competing interests

The authors have no non-financial, financial or patent related competing interests.

## Authors’ contributions

BJ grew and collected the plant material for the experiments. The work was planned by KL. The experimental work, data analysis and re-annotation of significantly altered transcripts were carried out by BJ and AJS together. BJ and KL wrote the manuscript. All authors have read and approved the manuscript.

## Supplementary Material

Additional file 1 **Table S1.** RT-qPCR results indicating the equal detection of the P25 transcript from the leaves, and the flowers of the three transgenic plants that were used in the microarray analysis. Click here for file

Additional file 2 **Table S2.** Normalized microarray data showing the up-regulated transcripts in leaves of P25 expressing plants. Click here for file

Additional file 3 **Table S3.** Overview of the up-regulated transcripts detected in the leaves of the P25 expressing plants. Click here for file

Additional file 4 **Table S4.** Normalized microarray data showing the up- and down-regulated transcripts in flowers of P25 expressing plants. Click here for file

Additional file 5 **Table S5.** Overview of the up-and-down regulated transcripts detected in the flowers of the P25 expressing plants. Click here for file

Additional file 6 **Table S6.** Up-regulated transcripts related to the protein synthesis and proteolysis in leaves of P25 expressing plants. Click here for file

Additional file 7 **Figure S1.** Up-regulation of signaling related genes (log2 value >1) is shown by using the MapMan software. The blue squares represent the number of genes up-regulated in different pathways that are involved in signaling. Click here for file

Additional file 8 **Figure S2.** 2D-polyacrylamide gel electrophoresis (2D-PAGE) analyses of the proteins separated from four separate sets of wild type (left panels) and P25-expressing transgenic plants (right panels) to visualize levels of various individual proteins. Gels are stained using coomassie blue. The molecular weight markers (loaded on the right side of the gels in the two upper panels, and on the left side of the gels in the two lower panels) represent weights of 250, 130, 100, 70, 55, 35, 25, 15 and 10 kDa. Equal amounts (250 μg) of the solubilized leaf protein samples were initially loaded to ach of the isoelectric focusing runs. Equal loadings were confirmed by 1D-SDS-PAGE gels (data not shown). Click here for file

Additional file 9 **Table S7.** List of genes that are up-regulated in leaves of all the P25, AC2 or HC-Pro VSRS expressing transgenic plants, as detected by microarray [21,49]. Click here for file

Additional file 10 **Table S8.** Overview of the transcripts that are up-regulated in all the P25, HC-Pro and AC2 VSRS expressing transgenic plants, as detected by microarray analysis [21,49]. Click here for file

Additional file 11 **Figure S3.** Over-presentation analysis using PageMan of leaves expressing P25, HC-Pro or AC2. The test was performed using Fisher’s exact test including FDR <0.05. Click here for file

Additional file 12 **Table S9.** RT-qPCR conditions and the sequences of the primers that were used to validate the microarray data. Click here for file
