# Supplementary material for: Organ-specific alterations in tobacco transcriptome caused by the PVX-derived P25 silencing suppressor transgene
Source: BMC Plant Biol. 2013 Jan 8;13:8. doi: 10.1186/1471-2229-13-8 (PMC3562197; doi:10.1186/1471-2229-13-8)
Supplement: Additional file 1 — Table S1. RT-qPCR results indicating the equal detection of the P25 transcript from the leaves, and the flowers of the three transgenic plants that were used in the microarray analysis. [file 1471-2229-13-8-S1.docx]

| Expression of P25 mRNA in transgenic plants measured using RT-qPCR. Numbers are indicating quantification cycle (C_q_) values. No PCR products were detected in wild type samples (N/A). | | | | | | |
| --- | --- | --- | --- | --- | --- | --- |
|  | **Leaf 1** | **Leaf 2** | **Leaf 3** | **Flower 1** | **Flower 2** | **Flower 3** |
| Samples (WT) | **WT1** | **WT2** | **WT3** | **WT1** | **WT2** | **WT3** |
| Technical replicate 1 | N/A | N/A | N/A | N/A | N/A | N/A |
| Technical replicate 2 | N/A | N/A | N/A | N/A | N/A | N/A |
| Technical replicate 3 | N/A | N/A | N/A | N/A | N/A | N/A |
| Sample (P25) | **P25 1** | **P25 2** | **P25 3** | **P25 1** | **P25 2** | **P25 3** |
| Technical replicate 1 | 18.57 | 18.55 | 18.37 | 20.38 | 18.52 | 19.64 |
| Technical replicate 2 | 18.43 | 19.07 | 18.61 | 20.64 | 18.75 | 19.72 |
| Technical replicate 3 | 18.78 | 19.27 | 18.69 | 20.78 | 19.09 | 19.79 |
| Mean | 18.593 | 18.963 | 18.556 | 20.60 | 18.786 | 19.716 |
| s.e. | 0.0830 | 0.1752 | 0.0785 | 0.0956 | 0.1351 | 0.0353 |

Three biological replicates of leaf and flower samples (WT and P25) are presented in the columns. Three technical replicates are also shown.
