# Supplementary material for: Organ-specific alterations in tobacco transcriptome caused by the PVX-derived P25 silencing suppressor transgene
Source: BMC Plant Biol. 2013 Jan 8;13:8. doi: 10.1186/1471-2229-13-8 (PMC3562197; doi:10.1186/1471-2229-13-8)
Supplement: Additional file 3 — Table S3. Overview of the up-regulated transcripts detected in the leaves of the P25 expressing plants. [file 1471-2229-13-8-S3.docx]

| **Table 2**. **A list of the up-regulated transcripts belonging in different functional groups in P25 leaves** | | |
| --- | --- | --- |
|  | **Total number of positive detections** | **Range of fold -change enhancement** |
| **BIOTIC STRESS RELATED** | **138** |  |
| Avr9/Cf-9 Elicitor or Hairpin elicited proteins | 21 | 2-12 x |
| Chitinases, endochitinases | 17 | 50 - 147 x |
| Disease resistance genes, putative | 21 | 2 - 42 x |
| Enhanced disease susceptibility 1, EDS1 | 2 | 4 - 8 x |
| Hypersensitive cell death associated proteins | 10 | 2 - 18 x |
| Miscellaneous | 7 | 3 - 38 x |
| Pathogenesis related proteins, 1a, 1b, 1c, 4B, R, PX, | 30 | 5 - 3061 x |
| Receptor protein kinases | 3 | 2 x |
| Retro element pol polyprotein | 5 | 2 - 10 x |
| SAR-related proteins | 19 | 10 - 280 x |
| TMV response-related gene product | 1 | 9 x |
| Tumor-related protein precursors, putative | 2 | 9 x |
|  |  |  |
| **CALCIUM BINDING, SIGNALLING AND PHOSPHARYLATION RELATED** | **123** |  |
| Ca-binding proteins | 18 | 2 - 26 x |
| Calmodulins, calmodulin binding proteins | 17 | 2 - 25 x |
| Kinase family proteins | 30 | 3 - 14 x |
| Light signaling: PAR, Photo assimilate-responsive protein | 5 | 8 - 424 x |
| MAP Kinase | 2 | 2 - 3 x |
| Map kinase phosphatase | 1 | 6 x |
| MAP kinases, MK1 and MAP3 | 7 | 2 - 8 x |
| Miscellaneous | 9 | 6 - 13 x |
| Receptor kinases, various | 32 | 2 - 26 x |
| Serine/threonine phosphatases | 2 | 4 x |
|  |  |  |
| **TRANSCRIPTION FACTORS AND SILENCING RELATED** | **87** |  |
| AGO2 (Argonaute 2), Putative | 1 | 4 x |
| DCL2 (DICER-LIKE 2) | 1 | 8 x |
| ERF-factors (ERF 1, 3 and 5) | 5 | 5 - 12 x |
| LEAFY 1 |  |  |
| Miscellaneous: transcription factors and RNA processing | 33 | 2 - 10 x |
| NAC and Nam-domains containing proteins | 13 | 2 - 41 x |
| RNA-directed RNA polymerase 1 | 1 | 4 x |
| SCARECROW gene regulator | 2 | 2-3 x |
| Transcriptional activator PTI5; Pathogenesis-related | 2 | 18 - 41 x |
| WRKY domain transcription factor family | 29 | 2 - 58 x |
|  |  |  |
| **SECONDARY METABOLITE RELATED** | **50** |  |
| 5-epi-aristolochene synthase | 18 | 7 – 199 x |
| REF/SRPP-like protein, isoprenoids | 3 | 4 - 5 x |
| Various secondary metabolite biosynthesis related | 30 | 2- 18 x |
|  |  |  |
| **PROTEIN SYNTHESIS, TARGETING, MODIFICATION AND DEGRADATION RELATED** | **148** |  |
| Hydrolases, various | 11 | 2 - 162 x |
| Nitrilases | 2 | 2 x |
| Proteases, endopeptidases and carboxypeptidases | 28 | 2 - 181 x |
| Protein disulfide isomerases | 2 | 8x |
| Protein kinases, various | 25 | 2 - 5 x |
| Protein phosphatases, various | 5 | 2 - 9 x |
| Protein secretion: ER lumen, vesicles, peroxisomes, nucleus | 19 | 2 - 33 x |
| Proteinase inhibitors | 4 | 3 - 99 x |
| Transcription initiation (sigma factors) | 3 | 25 – 58.8x |
| Translation machinery and initiation - related proteins | 9 | 2 - 33 x |
| Ubiquitination related, various | 40 | 2 - 70 x |
|  |  |  |
| **DEVELOPMENTAL, CELL CYCLE AND CYTOSCELETON RELATED** | **22** |  |
| Actin | 1 | 5 x |
| Actin depolymerizing factors | 2 | 2 x |
| DYNAMIN-LIKE 1E, putative | 1 | 6 x |
| Embryo-abundant protein, putative | 7 | 4 - 5 x |
| RabGAP/TBC/microtubule-associated protein | 1 | 2 x |
| Senescence-associated proteins | 3 | 2 - 3 x |
| Spindle disassembly related protein CDC48 | 1 | 2 x |
| Storage proteins (patatin, cupin) | 3 | 4 - 10 x |
| WD-repeat protein /TTG1 (TRANSPARENT TESTA GLABRA 1) | 1 | 2 x |
| Yippee-like protein related cluster | 2 | 48 - 107 x |
|  |  |  |
| **HORMONE METABOLISM RELATED** | **37** |  |
| Auxin responsive proteins | 6 | 2 - 7 x |
| Auxin independent proteins | 2 | 2 x |
| Ethylene synthesis | 7 | 3 - 95 x |
| Ethylene induced / responsive elements | 4 | 6 - 40 x |
| Gibberellin related: receptors, oxidases | 5 | 4 - 9 x |
| Lipoxygenases | 8 | 3 - 8 x |
| Multiprotein bridging factor 1, putative | 5 | 2 - 31 x |
|  |  |  |
| **LIPID METABOLISM AND MEMBRANE ASSOCIATED** | **41** |  |
| Enoyl-CoA hydratase / Fatty oxidation complex | 7 | 2 - 4 x |
| Lipid transfer protein 1 (e.g. LTP1) | 6 | 2 - 94 x |
| Membrane & lipid modification activities | 9 | 2 - 11 x |
| Membrane proteins, various | 15 | 2 - 12 x |
| Phospholipases | 4 | 2 - 7 x |
|  |  |  |
| **GLUTATHIONE S-TRANSFERASES** | **44** |  |
| Glutathione S-transferase | 42 | 2 - 80 x |
| MAPEG (Membrane Associated, Eicosanoid and Glutathione metabolism) | 2 | 2 - 3 x |
|  |  |  |
| **ABIOTIC STRESS** | **41** |  |
| Development and cell death domain + B protein | 3 | 4 - 6 x |
| Heat shock protein 17.9 | 26 | 2 - 7 x |
| Oxidative stress response | 4 | 11 x |
| Salt, drought, cold stress responsive proteins | 8 | 2 - 73 x |
|  |  |  |
| **NUCLEOTIDE BINDING AND PROCESSING PROTEINS** | **31** |  |
| AAA ATPase | 12 | 3 - 70 x |
| ATP binding protein, putative | 6 | 2 - 9 x |
| GTP binding protein RAP, RAS, Small G-proteins | 8 | 2 - 3 x |
| Nucleotide processing | 5 | 2 - 4 x |
| **CELL WALL RELATED** | 26 |  |
| Acetylglucosaminyltransferase, putative | 2 | 3 x |
| Cell wall proteins | 6 | 3 - 20 x |
| Glucan endo-1,3-beta-glucosidase | 6 | 8 - 66 x |
| Hydroxy proline-rich glycoprotein | 2 | 2 - 7 x |
| Pectin methylesterase inhibitor family protein, putative | 2 | 2 - 20 x |
| S-adenosylmethionine decarboxylase | 1 | 3 x |
| Xyloglucan endotransglucosylase-hydrolase | 9 | 3 - 14 x |
|  |  |  |
| **CYTOCHROME P450** | **21** |  |
| Cytochrome p450 related | 21 | 2 - 63 x |
|  |  |  |
| **SUGAR AND AMINO ACID METABOLISM** | **138** |  |
| Reductases, oxidoreductases, thioredoxins, various | 29 | 2 - 39 x |
| Glycosyltransferases, various | 20 | 2 - 30 x |
| Dehydrogenases, various | 27 | 2 - 24 x |
| Decarboxylases, kinases | 8 | 2 - 10 x |
| Aconitases, epimerases, invertases, amylases, fructosidases, mutases, hydrolases, isomerases | 21 | 2 - 23 x |
| Peroxidases | 17 | 2 - 14 x |
| Oxidases | 4 | 12 - 2 x |
| Mitochondrial electron transfer | 5 | 2 - 7 x |
| Amino-, acyl- , acetyltransferases, Thiolases | 11 | 2 - 17 x |
| Miscellaneous | 6 | 3 - 2 x |
|  |  |  |
| **TRANSPORT RELATED** | **81** |  |
| Amino acid and Oligopeptide transporter proteins | 17 | 2 - 25 x |
| Ion channels | 8 | 3 - 26 x |
| Multidrug resistance proteins /ABC transporters | 13 | 3 - 8 x |
| Phosphate, sulphate, ammonium, metal, nitrate transport | 21 | 2 - 17 x |
| Sugar transporters | 7 | 2 - 7 x |
| Transport related, miscellaneous | 15 | 2 - 17 x |
|  |  |  |
| **DNA-BINDING PROTEINS** | **11** |  |
| DNA binding protein, putative | 4 | 2 - 9 x |
| Drm3-like protein | 1 |  |
| Ku70-like protein | 1 | 2 x |
| Microsatellite DNA | 4 | 3 - 4 x |
| Sister chromatid cohesion 1 protein, low query coverage | 1 |  |
|  |  |  |
| **FUNCTIONS NOT ASSIGNED** | **39** |  |
| miscellaneous | 39 | 2 - 44 x |
|  |  |  |
| **UNIDENTIFIED** | **269** | 2 - 120 x |
| DUF domains | 12 |  |
| others | 257 |  |

| The table represents the total number of detections of transcripts that were up-regulated more than two-fold in the P25-expressing plants. Statistical significance was tested by using student t-test (p<0.05) with the BH false discovery rate of 5%. |
| --- |
|  |
