# Supplementary material for: Organ-specific alterations in tobacco transcriptome caused by the PVX-derived P25 silencing suppressor transgene
Source: BMC Plant Biol. 2013 Jan 8;13:8. doi: 10.1186/1471-2229-13-8 (PMC3562197; doi:10.1186/1471-2229-13-8)
Supplement: Additional file 5 — Table S5. Overview of the up-and-down regulated transcripts detected in the flowers of the P25 expressing plants. [file 1471-2229-13-8-S5.docx]

| **Table 5**. **A list of up and down-regulated transcripts belonging in different functional groups in P25 flowers.** | | |
| --- | --- | --- |
|  | **Total number of genes** | **Range of fold -change** |
| **PHOTOSYNTHESIS, SUGAR AND SIGNALING RELATED** | **5** |  |
| Receptor-like protein kinase | 1 | 0.43 x |
| Photosynthesis related  Sugar signaling | 3  2 | 0.28 – 4.0 x  2.6 – 8.8 x |
|  |  |  |
| **PROTEIN RELATED** | **10** |  |
| Amino acid metabolism related | 3 | 0.18- 0.4 x |
| Metallothionein-like protein type 2 | 1 | 2.87 x |
| NEK6 (Never in mitosis, gene A) | 1 | 0.35 x |
| Ribosomal protein S10-like protein | 1 | 2.31 x |
| Various proteases and protease inhibitors | 4 | 2.2 - 6.5 x |
|  |  |  |
| **SECONDARY METABOLISM** **TRANSCRIPTION FACTORS RELATED** | **3** |  |
| GATA transcription factors  Vetispiradiene synthase | 1  2 | 2.78 x  3.28 – 4.3 x |
|  |  |  |
| **MISCELLANEOUS AND UNKNOWN** | **12** |  |
| Miscellaneous and unknown proteins | 12 | 0.37 - 8.5 x |
|  |  |  |
| **BIOTIC AND ABIOTIC STRESS RELATED GENES** | **34** |  |
| Epoxide hydrolase | 3 | 2.8 – 3.7 x |
| Glutathione S-transferase TAU 19 | 1 | 2.82 x |
| Various –PR related, SAR, chitinases, transposons and LRR proteins | 30 | 0.44 – 8.3 x |
| The table represents the total number of detections of transcripts that were both up and down-regulated more than two-fold in the P25-expressing plants. Statistical significance was tested by using student t-test (p<0.05. | | |
|  | | |
