# Supplementary figures and images for: Organ-specific alterations in tobacco transcriptome caused by the PVX-derived P25 silencing suppressor transgene
Source: BMC Plant Biol. 2013 Jan 8;13:8. doi: 10.1186/1471-2229-13-8 (PMC3562197; doi:10.1186/1471-2229-13-8)

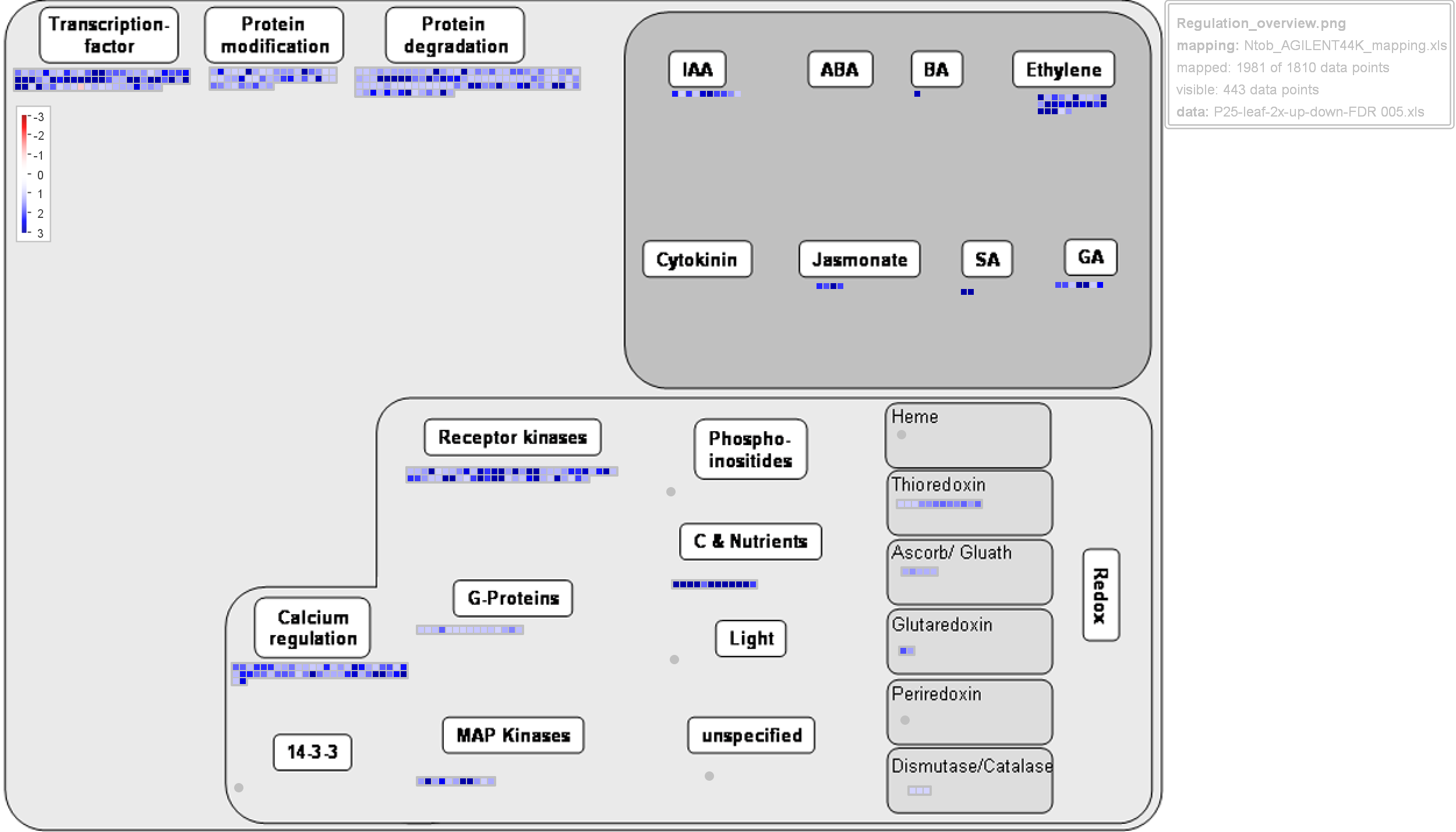

Supplement: Additional file 7 — Figure S1. Up-regulation of signaling related genes (log2 value >1) is shown by using the MapMan software. The blue squares represent the number of genes up-regulated in different pathways that are involved in signaling. [file 1471-2229-13-8-S7.png]

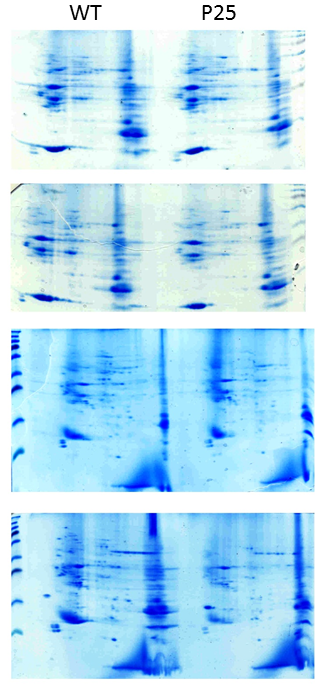

Supplement: Additional file 8 — Figure S2. 2D-polyacrylamide gel electrophoresis (2D-PAGE) analyses of the proteins separated from four separate sets of wild type (left panels) and P25-expressing transgenic plants (right panels) to visualize levels of various individual proteins. Gels are stained using coomassie blue. The molecular weight markers (loaded on the right side of the gels in the two upper panels, and on the left side of the gels in the two lower panels) represent weights of 250, 130, 100, 70, 55, 35, 25, 15 and 10 kDa. Equal amounts (250 μg) of the solubilized leaf protein samples were initially loaded to ach of the isoelectric focusing runs. Equal loadings were confirmed by 1D-SDS-PAGE gels (data not shown). [file 1471-2229-13-8-S8.png]
