# Supplementary material for: Organ-specific alterations in tobacco transcriptome caused by the PVX-derived P25 silencing suppressor transgene
Source: BMC Plant Biol. 2013 Jan 8;13:8. doi: 10.1186/1471-2229-13-8 (PMC3562197; doi:10.1186/1471-2229-13-8)
Supplement: Additional file 10 — Table S8. Overview of the transcripts that are up-regulated in all the P25, HC-Pro and AC2 VSRS expressing transgenic plants, as detected by microarray analysis [21,49]. [file 1471-2229-13-8-S10.docx]

| **Table 6**. **Comparison of microarray data indicating the commonly altered up-regulated transcripts in P25-, HcPro- and AC2-expressing transgenic plants** | | |
| --- | --- | --- |
| **Functional classification** | **Total number of detections** | **Range of fold -change enhancement** |
|  |  |  |
| **P25-HcPro common upregulated genes** | 29 |  |
| Cell wall, transporters and transferase related | 3 | 2 - 3 x |
| Hormone metabolism | 2 | 5-7 x |
| Protein synthesis, modification and degradation related | 6 | 3 - 6 x |
| Secondary metabolism | 4 | 10 -38 x |
| Signaling related | 1 | 2 x |
| Stress and ROS related | 5 | 2 - 214 x |
| Transcription factors | 3 | 3 - 5 x |
| Unknown | 5 | 2 - 45 x |
|  |  |  |
| **P25-AC2 common upregulated genes** | 112 |  |
| Amino acid and proteins related | 13 | 2 - 70 x |
| Cell cycle, development and cell wall related | 7 | 2 - 106 x |
| Hormones related | 7 | 4 - 78 x |
| Hydrolyses | 2 | 2 - 6 x |
| Interesting and miscellaneous | 2 | 3 - 44 x |
| Polyamine and secondary metabolism related | 4 | 2 - 13 x |
| ROS related | 11 | 2 - 63 x |
| Signaling | 8 | 3 - 56 x |
| Stress related | 20 | 3 - 102 x |
| Sugar metabolism and e-transport related | 6 | 2 - 7 x |
| Transcription related | 11 | 3 - 15 x |
| Transporters related | 9 | 2 - 14 x |
| Unknown | 12 | 2 - 12 x |
|  |  |  |
| The table represents the total number of positive detections that were commonly altered more than two-fold in P25, HcPro and AC2 VSR expressing transgenic plants. Statistical significance was tested by using student t-test (p<0.05) | | |
