# Supplementary material for: Organ-specific alterations in tobacco transcriptome caused by the PVX-derived P25 silencing suppressor transgene
Source: BMC Plant Biol. 2013 Jan 8;13:8. doi: 10.1186/1471-2229-13-8 (PMC3562197; doi:10.1186/1471-2229-13-8)
Supplement: Additional file 12 — Table S9. RT-qPCR conditions and the sequences of the primers that were used to validate the microarray data. [file 1471-2229-13-8-S12.docx]

**Supplemental table 9. Overview of RT-qPCR conditions according to the BIOMED central MIQE precise file format**

| **Sample/Template** | **details** |
| --- | --- |
| Source | Wild type and P25 expressing transgenic tobacco plants |
| Method of preservation | Liquid N2 and storage at -80 C |
| Storage time (if appropriate) | Stored frozen less than six months |
| Handling | frozen |
| Extraction method | Trisure (Bioline,UK) |
| RNA: DNA-free | DNAse I treatment (Promega RQ1 RNase free-DNAseI) |
| Concentration | Nanodrop |
| RNA: integrity | Agilent\2100 bioanalyzer |
| Inhibition-free | N/A |
| **Assay optimisation/validation** |  |
| Accession number | N/A |
| Amplicon details | See below |
| Primer sequence | see below |
| *Probe sequence** | No probes |
| *In silico* | Primer3 program used for designing primers |
| empirical | 0.2uM primer concentration/55 -60 C annealing temperature |
| Priming conditions | oligo-dT used for cDNA synthesis |
| PCR efficiency | Tested in conventional PCR |
| Linear dynamic range | Variable for different amplicons |
| Limits of detection | N/A |
| Intra-assay variation | N/A |
| **RT/PCR** |  |
| Protocols | See methods section, 3ul of cDNA with 3.3ng/ul concentration, 25 ul total volume |
| Reagents | Fermentas, EPO441-Reverse transciptase; Fermentas,K0242- Maxima SYBR Green/Fluorescein RT-qPCR Master Mix (2X) |
| Duplicate RT | 3 biological replicates S.E shown in table 1 |
| NTC | Melt curves checked and clean |
| NAC | Variable for different amplicons |
| Positive control | Same reference gene used in different runs |
| Data analysis |  |
| Specialist software | Bio-RAD iQ5 |
| Statistical justification | 3 biological replicates |
| Transparent, validated normalisation | Normalized with the reference gene |

**Primers used in the qRT-PCR for validating the microarray expression profile**

**Primers for differentially expressed genes**

EH617029: WRKY transcription factor-30

FORWARD PRIMER 5’-TGTGGATGTGATCACCAGAA-3’

REVERSE PRIMER 5’-TGAATTTCACTCGCAGCTTG-3’

EB438730: Dicer-2 like

FORWARD PRIMER 5’-ACCTGAGATTCCACGATGCT-3’

REVERSE PRIMER 5’-ACACCGTCAGCTTCAACCTC-3’

EH620111: Pathogenesis-related protein 1B precursor

FORWARD PRIMER 5’-CAT GCC CAA AAC TCT CAA CA-3’

REVERSE PRIMER 5’-CCT AGC ACA TCC AAC ACG AA-3’

EB681684: ABIL1 protein

FORWARD PRIMER 5’-AACTTCGGATTGATGGCTTG-3’

REVERSE PRIMER 5’-TTAGCTTTCGAGCCTCCTGA-3’

TA12713_4097: Chloroplast light-harvesting complex II protein (Lhcbm6)

FORWARD PRIMER 5’-TCT CCG AGC AAA CTC CAT CT-3’

REVERSE PRIMER 5’-TTC AAT TGG TCC CTT TCC TG-3’

EG650355: B-1, 3 glucanase, putative

FORWARD PRIMER 5’-GTGAAGCGGGGAAGGATAAT-3’

REVERSE PRIMER 5’-GCACGCCCTTTCTTCTGATA-3’

Z11563: Acidic endochitinase precursor

FORWARD PRIMER 5’-GGC CAA AAT CCA GTG CTA AA -3’

REVERSE PRIMER 5’-GAA AGT GCC CCA TTT AAC CA -3’

**Primers for reference genes**:

EB450395: ARPC3 (actin-related protein C3) protein like

FORWARD PRIMER 5’-CGC CGG TGA AAT TAC CTC T -3’

REVERSE PRIMER 5’-AAG TTC TGC AGC CTT CAA GC -3’

AM833694: DCL 1 (Dicer like 1)

LEFT PRIMER 5’-TCA TTG GGA GTG CAA TCA AC -3’

RIGHT PRIMER 5’-TAT CTT GGC ACC AGT GAA CG -3’
